# Bacterial Morphotypes as Important Trait for Uropathogenic *E. coli* Diagnostic; a Virulence-Phenotype-Phylogeny Study

**DOI:** 10.3390/microorganisms9112381

**Published:** 2021-11-18

**Authors:** Manuel G. Ballesteros-Monrreal, Margarita M. P. Arenas-Hernández, Edwin Barrios-Villa, Josue Juarez, Maritza Lizeth Álvarez-Ainza, Pablo Taboada, Rafael De la Rosa-López, Enrique Bolado-Martínez, Dora Valencia

**Affiliations:** 1 Departamento de Ciencias Químico-Biológicas, Universidad de Sonora, Hermosillo C.P. 83000, Sonora, Mexico; manuel.ballesteros@unison.mx (M.G.B.-M.); maritza.alvarez@unison.mx (M.L.Á.-A.); 2 Posgrado en Microbiología, Centro de Investigación en Ciencias Microbiológicas, Instituto de Ciencias, Benemérita Universidad Autónoma de Puebla, Ciudad Universitaria, Puebla C.P. 72570, Pue, Mexico; margarita.arenas@correo.buap.mx; 3 Departamento de Ciencias Químico-Biológicas y Agropecuarias, Universidad de Sonora, Caborca C.P. 83621, Sonora, Mexico; edwin.barrios@unison.mx (E.B.-V.); rafael.delarosa@unison.mx (R.D.l.R.-L.); 4 Departamento de Física, Universidad de Sonora, Hermosillo C.P. 83000, Sonora, Mexico; josue.juarez@unison.mx; 5 Grupo de Física de Coloides y Polímeros Departamento de Física de Partículas, Universidad de Santiago de Compostela, C.P. 15782 Santiago de Compostela, Spain; pablo.taboada@usc.es

**Keywords:** urinary tract infection, UPEC morphotypes, UPEC virulence

## Abstract

Urinary tract infections (UTIs) belong to the most common pathologies in Mexico and are mainly caused by Uropathogenic *Escherichia coli* (UPEC). UPEC possesses a wide diversity of virulence factors that allow it to carry out its pathogenesis mechanism in the urinary tract (UT). The development of morphotypes in UT represents an important feature of UPEC because it is associated with complications in diagnosis of UTI. The aim of this study was to determine the presence of bacterial morphotypes, virulence genes, virulence phenotypes, antibiotic resistant, and phylogenetic groups in clinical isolates of UPEC obtained from women in Sonora, Mexico. Forty UPEC isolates were obtained, and urine morphotypes were observed in 65% of the urine samples from where *E. coli* was isolated. Phylogenetic group B2 was the most prevalent. The most frequent virulence genes were *fimH* (100%), *fliCD* (90%), and *sfaD/focC* (72%). Biofilm formation (100%) and motility (98%) were the most prevalent phenotypes. Clinical isolates showed high resistance to aminoglycosides and β-lactams antibiotics. These data suggest that the search for morphotypes in urine sediment must be incorporated in the urinalysis procedure and also that clinical isolates of UPEC in this study can cause upper, lower, and recurrent UTI.

## 1. Introduction

Urinary tract infections (UTIs) are one of the most common pathologies in Mexico with more than 4 million cases reported each year [1,2]. Although UTIs are common in both males and females, the prevalence is higher in women (>70%). In this regard, it is estimated that 50% of all women worldwide will present at least one episode of UTI in their lives, and 30% of this population will experience recurrent episodes [3,4].

The etiology of UTI is varied; however, the main causative pathogen of this condition is uropathogenic *Escherichia coli* (UPEC) [3,4]. In contrast to other *E. coli* pathotypes, UPEC does not possess a specific virulence profile, but its virulence genes are mainly associated with characteristics such as adherence, motility, iron capture, and toxigenicity. These virulence features allow UPEC to adapt and carry out successfully its pathogenesis mechanism in the urinary tract [5,6,7]. In this sense, one of the most important virulence traits of UPEC is its adherence capacity: it is known that the fimbrial adhesin FimH allows the pathogen not only to adhere to the bladder, but also favors its internalization in the target cell forming biofilm-like communities, denominated intracellular bacterial communities (IBC), which are associated with persistence in the urinary tract, antimicrobial resistance, and recurrent UTI [4,8]. In addition to IBC, UPEC can also form biofilm and filamentous bacteria in the urinary tract, which are implicated in antimicrobial resistance and immune evasion. In the clinical environment, the presence of these, also called bacterial morphotypes, in urinary sediments is important since they could be used as an additional valuable tool in the microbiological diagnosis of UTI due to UPEC [4,9,10,11].

On the other hand, it is known that *Escherichia coli* can be phylogenetically classified into seven phylogenetic groups (A, B1, B2, C, D, E, and F). Among these groups, B2 and D are those associated with pathogenic strains for humans, while groups A and B1 are related to both commensal and antibiotic resistance strains [12,13]. However, a high prevalence of UPEC belonging to phylogenetic groups considered to be non-pathogenic has been observed causing disease, which besides their multidrug resistance, also show a significant number of virulence associated genes [14,15,16].

Urinary tract infections represent the third most common cause of morbidity in Mexico. Despite its importance, there is little evidence focused on UPEC and its virulence characteristics. The knowledge of the prevalent virulence features in clinical isolates of UPEC will allow us to better understand its pathogenesis mechanisms and its possible implication in the improvement of the diagnosis and treatment of UTI. In this context, the aim of this work is to determine the more prevalent phylogenetic groups, virulence genes, virulence phenotypes, antibiotic resistant, and bacterial morphotypes of clinical isolates of UPEC recovered from women in Mexico.

## 2. Materials and Methods

### 2.1. Urine Samples Collection

Urine samples were collected from outpatients assisted in a public hospital in Sonora, Mexico, following aseptic directions. Male patients, children, and those who refused to give consent were not included in the study. Clinical data (age, signs, and symptoms, UTI recurrence, antibiotics treatments, and functional or morphological abnormalities in urinary tract) were collected in a survey. Patient data were maintained under anonymity.

### 2.2. Urinalysis and Detection of UPEC Morphotypes in Urine Sediment

The obtained urine samples were examined using URISPIN-U120 (Spinreact, Girona, Spain) with URIN-10 (Spinreact, Girona, Spain) dipsticks. For UPEC morphotypes detection, 10 mL of urine were centrifuged for 10 min at 400× *g*. The urine sediment was examined microscopically using Sternheimmer-Malbin stain. Samples were considered as positive for presence of morphotype if adherence, IBC, or filamentous bacteria were observed. According to previously proposed criteria and morphologic characteristics, adherence phenotype was considered positive when bacteria attached to epithelial cells were observed, while detection of dark-pink staining cells with suggestive images of intracellular bacteria and filamentous bacteria was considered as positive for IBC and *E. coli* filamentation, respectively [11,17].

### 2.3. Urine Cultures and Biochemical Identification of Obtained Bacterial Isolates

Urine samples were inoculated (1 µL with a sterile loop) on MacConkey agar and Mannitol-Salt agar for microbiological analysis. For CFU/mL count, samples were seeded on Trypticase Soy Agar (TSA) using a calibrated loop (0.001 mL). If morphotypes were observed, 10 mL of urine sample were vortexed for 1 min to release intracellular bacteria and seeded on additional TSA plate for CFU counts. Cultures were incubated for 18–24 h at 37 °C.

Uropathogens were identified by IMViC tests (indole, methyl red, vogues-Proskauer, and citrate production). In addition, urease, lysine decarboxylase, and ornithine decarboxylase production were included. Clinical isolates that were not identified as *E. coli* were reported but were not considered in this study.

If the patients had symptoms of UTI or bacterial morphotypes were observed in urine sediment, less than 10^5^ CFU/mL were considered as positive for urine culture [18].

### 2.4. DNA Extraction

Bacterial DNA was obtained by alkaline lysis, according to protocols previously reported [19].

### 2.5. Molecular Identification of E. coli

Clinical isolates were confirmed by polymerase chain reaction (PCR) using primers for the *ybbW* gene that encodes for an allantoin receptor, which is highly specific for *E. coli* [20]. The PCR product was observed by electrophoresis on a 2% agarose gel in 1× TAE buffer stained with GelStarTM Stain (Lonza, Morristown, NJ, USA).

### 2.6. Identification of Phylogenetic Groups

The method described by Clermont et al. 2013 was used to identify the phylogenetic group. This method is based on detection of *arpA*, *chuA*, *yjaA,* and *TspE4.C2* genes by using a quadruplex PCR [13].

### 2.7. Genotypic Characterization of UPEC Isolates

Virulence associated genes were identified by multiplex polymerase chain reaction (mPCR). Eighteen genes were investigated in six multiplex PCR: mPCR 1 (Adherence associated genes): *fimH* (type 1 pilus adhesin), *sfaD/focC* (S and Dra fimbriae), *papG-II* (type P pilus adhesin allele 2), and *papC* (type P pilus chaperone); mPCR 2 (Motility and toxigenicity associated genes): *fliCD* (flagella), *sat* (autotransporter secreted toxin); mPCR 3 (Immune evasion and toxigenicity associated genes): kpsM (capsule) and *hlyA* (α-hemolysin); mPCR 4 (immune evasion and toxigenicity associated genes): *traT* (serum resistance protein), *agn43* (43 antigen), *vat* (vacuolating autotransporter toxing), *cnf-1* (necrotizing cytotoxic factor); mPCR 5 (iron uptake associated genes): *fyuA* (ferric yersiniabactin uptake receptor), *iucD* (aerobactin), *iroN* (salmocheline receptor); mPCR 6 (iron uptake associated genes): *iutA* (aerobactin receptor), *feoB* (ferrous iron transporter), *iha* (irgA homologue Adhesin/enterobactin receptor). *E. coli* CFT073, *E. coli* ATCC 25922, and *E. coli* GAGI were used as a positive control for all evaluated genes. Control strains *E. coli* CFT073 and *E. coli* GAGI were kindly donated by Ph.D. Margarita MP Arenas-Hernández from Centro de Investigación en Ciencias Microbiológicas, Instituto de Ciencias, Benemérita Universidad Autónoma de Puebla. Primer sequences, length of their amplified products, and annealing temperature (Tm °C) are listed in Table 1.

Each PCR reaction was performed using a master mix containing 2 µL of buffer solution, 0.5 µL of a dNTP mixture (10 mM each one), 1.5 µL of 25 mM MgCl_2_, 0.5 µL of each primer (10 µM), 0.1 µL of GoTaq^®^ Flexi DNA Polymerase (Promega), 1.5 µL [50–75 ng] of template DNA, and necessary distilled water to obtain a final volume of 15.5 µL. Reactions were performed in ProFlex™ PCR System (Thermo Fisher, Waltham, MA, USA). Conditions implemented were: One cycle at 95 °C for 4 min, 35 cycles at 95 °C for 1 min and 10 s, 72 °C for 1 min, and 1 cycle at 72 °C for 10 min. Annealing temperature (Tm °C) and times of reactions were 54 °C for 1 min and 10 s (mPCR 1), 60 °C for 1 min (mPCR 2), 58 °C for 1 min (mPCR 3 and mPCR 4), and 60 °C for 45 s (mPCR 5 and mPCR 6). PCR products were observed by electrophoresis on a 2% agarose gel in 1× TAE buffer stained with GelStarTM Stain (Lonza, USA).

### 2.8. Phenotypic Characterization of UPEC Isolates

#### 2.8.1. Motility Test

A 24 h pre-culture of the bacterial isolate was obtained on nutrient agar. For motility detection, tubes with semisolid agar (SIM) were used, UPEC isolates were inoculated with a single stab of an inoculating loop and incubated for 18–24 h at 37 °C. A positive phenotype showed growth away from the stab line of inoculation, evidenced by turbidity. While a negative result is defined by confined growth in the stab line. *E. coli* CFT073 (positive phenotype) and *E. coli* EDL 933 (negative phenotype) were used as a control in each experiment.

#### 2.8.2. α-Hemolysin Production

Twenty-five microliters of a 24 h pre-culture in Luria-Bertani broth (LB) of each UPEC isolate were inoculated in a previously created well in 5% sheep blood agar plate and incubated for 24 h at 37 °C. The presence of hemolysis around the inoculated well was considered as a positive phenotype. *Escherichia coli* CFT073 was used as a positive control, while culture media without bacteria was used as a negative control [14].

#### 2.8.3. Biofilm Formation Assay

The ability to produce biofilm was determined following established protocols with slight modifications [21]. A 24 h preculture of UPEC in Mueller-Hinton Broth supplemented with 1% of glucose was diluted 1:100. Five hundred µL of this dilution were deposited in a microtube and incubated for 24 h at 37 °C. After incubation, planktonic bacteria were removed by gently aspired using a micropipette, washed twice with phosphate buffer saline pH 7.2 (PBS), and fixed with 500 µL of sodium acetate (2% *w*/*v*) for 20 min. After that, the microtube was washed again with PBS and stained with 500 µL of crystal violet (0.5% *w*/*v*) for 15 min. The remaining crystal violet was removed, and the microtube was washed with water until clearance. Finally, 500 µL of acetic acid (30% *v*/*v*) were used to resuspend the crystal violet, and 100 µL were deposited in a polystyrene 96 well plate for optical density reading at 550 nm with an ELISA plate reader (Multiskan EX, ThermoLabSystem, Waltham, MA, USA). *E. coli* ATCC 25,922 was used as a control. Previously established criteria were used to grade the isolates in different biofilm-producing groups based on the optical density (OD) obtained: OD (problem isolate) ≤ ODc (control strain) = no biofilm producer, ODc < OD ≤ 2x ODc = moderate biofilm producer, 4x ODc < OD = strong biofilm producer [22]

#### 2.8.4. Capsule Production

The capsule phenotype was identified using Anthony’s stain method. One bacterial colony of each isolate from a 48 h pre-culture on LB agar was deposited onto a glass slide, one drop of physiological solution was added, mixed, and dried at room temperature. The sample was stained with 1% of crystal violet for 1 min and washed with a 20% (*w*/*v*) copper sulphate solution and observed by light field microscopy. The presence of a faint blue halo around a purple cell was indicative of positive capsule phenotype. *Escherichia coli* CFT073 was used as a positive control [23].

#### 2.8.5. Adherence Assay

Twenty clinical isolates with the higher number of adherence associated genes (*fimH*, *fliCD*, *sfaD/focC*, *papG-II*, *kpsM*, *iha*, *papC*, and *agn43*) (Supplementary Material Table S1), with biofilm- or capsule-producing phenotypes, and morphotypes in urinary sediment were randomly selected. Additionally, 3–5 clinical isolates from each phylogenetic group were included. HeLa cells were seeded on culture plates in Dulbecco’s Modified Eagle Medium (DMEM) (SIGMA) supplemented with 5% fetal bovine serum (FBS) (GIBCO) and incubated at 37 °C in 5% CO_2_ until sub-confluence. Six-well polystyrene plates with coverslips were used, and a cellular suspension of 5 × 10^4^ cells/mL was prepared in 2 mL DMEM supplemented with 10% FBS without antibiotics. Plates were then incubated overnight at 37 °C in 5% CO_2_. HeLa cells monolayers were washed with sterile PBS. After washing, 2 mL of fresh DMEM supplemented with 10% FBS were added to each well. From an 18–24 h pre-culture in Brain Heart Infusion Broth (BHI) of the problem bacterial isolate, an adjustment was made to 0.5 on the McFarland scale in DMEM and 15 µL of this suspension was placed in contact with the HeLa cells (30:1, Bacteria: HeLa). The plate was then incubated at 37 °C and 5% CO_2_ for 3 h. Then, the cells were washed with PBS to remove unattached bacteria, fixed with methanol, stained with Giemsa, washed with PBS three times, and finally, coverslips were removed and mounted on a slide for microscopic observation. *Escherichia coli* strain EDL 933 (EHEC) was used as a positive control. Each clinical isolate was evaluated in three independent experiments by triplicate. Total HeLa cells and adherent bacteria were counted in 10 fields at 40X objective. The results are expressed as the average number of adherent bacteria. Isolates were classified as low adherent- (≤3 bacteria/HeLa cell), moderately adherent- (4–7 bacteria/HeLa cell), and highly adherent- (≥8 bacteria/HeLa cell) [24,25].

#### 2.8.6. Antibiotic Resistance

Twenty-one antibiotics from twelve categories were tested by disk diffusion method following directions stablished in the Clinical and Laboratory Standards Institute (CLSI, 2021). Antibiotic tested in this study were: Aminoglycosides: Amikacin (AMK, 30 µg), Gentamicin (GM, 10 µg); Fluoroquinolones: Ciprofloxacin (CIP, 5 µg), Levofloxacin (LVX, 5 µg), Norfloxacin (NOR, 10 µg); Sulphas: Cotrimoxazole (TSX, 1.25/23.75 µg); Nitrofurans: Nitrofurantoin (MAC, 300 µg); Penicillin: Ampicillin (AMP, 10 µg); 2th–4th generation cephalosporins: Cefoxitin (CX, 30 µg), Cefuroxime (CX, 30 µg), Ceftazidime (CFZ, 30 µg), Cefotaxime (CTX, 30 µg), Ceftriaxone (CRO, 30 µg), Cefepime (FEP, 30 µg); Monobactams: Aztreonam (ATM, 30 µg); β-lactam combination agents: Amoxicillin/Clavulanate (AMC, 20/10 µg), Ampicillin/Sulbactam (10/10 µg); Tetracyclines: Tetracycline (TE, 30 µg); Carbapenems: Imipenem (IMP, 10 µg), Meropenem (MEM, 10 µg), and Ertapenem (ETP, 10 µg). According to number of antibiotic categories, clinical isolates were classified as no multidrug resistant (NMDR, non-susceptible to less than 3 antibiotic categories), multidrug resistant (MDR, non-susceptible to at least 1 agent in 3 antimicrobial categories), extensively resistant (XDR, non-susceptible to at least 1 agent in all but two or fewer categories), or pandrug resistant (PDR, non-susceptible to all evaluated antimicrobial agents). Non-susceptible is defined as a clinical isolate which had resistant or intermediate resistant phenotype to an antimicrobial agent, according to Mayiorakos considerations [26].

### 2.9. Statistical Analysis

The results were analyzed using ANOVA Tukey’s multiple comparisons test, Pearson correlation coefficient, and Fisher’s exact test, using GraphPad Prims 6.04 software for Windows, GraphPad Software, La Jolla, CA, USA, www.graphpad.com, (accessed on 26 October 2021). The level of significance was considered as a *p* value ≤ 0.05. For Pearson correlation test, we statistically analyzed all genotypes, phenotypes, and phylogenetic groups against another; *r* values were obtained, and *p* value was confirmed with Fisher’s exact test or Chi-square.

### 2.10. Ethic Statements

The protocol for this study was approved by the ethical committee from Universidad de Sonora (CEI-UNISON) (Registry number 07.2019, 12 March 2019).

## 3. Results

### 3.1. Clinical Characteristics of Adult Women with UTI

Ninety-eight urine samples were analyzed. Eighty-five were included in this study, whereas the remaining were obtained from men, children, or those who refused to give consent and were not included. Forty (47%) patients had UTI according to urine culture results.

The included patients (*n* = 85) average age was 47 years old, ranging from 19 to 80 years. No statistical significance was observed between average age of patients with UTI and without it (*p* < 0.05). Fifty-four (63%) had co-morbidities and UTI predisposing conditions, with diabetes, hypertension, hypothyroidism, renal failure, and previous diagnosis of UTI being the most frequent. Nevertheless, no statistically significant association of these conditions with UTI was observed. On the other hand, in urinalysis, statistically significant differences were observed for the higher prevalence of positive leukocyte esterase, pyuria, and bacteriuria in urine samples from patients with UTI vs. patients without UTI (Table 2a).

Twenty-five (62%) patients with UTI (*n* = 40) were symptomatic, with renal pain being the most frequent clinical symptom (73%). Fourteen of these patients (56%) showed lower urinary tract symptoms, dysuria being the most prevalent (100%). Other symptoms were fetid urine (12%) and urinary frequency (16%). More than one symptom was observed in 15 (60%) patients. On the other hand, 15 (38%) patients, with positive urine culture, were asymptomatic.

Additionally, twenty-eight (70%) of the patients with positive urine cultures reported recurrent UTI with two or more episodes each year. Ten (36%) of this patient’s group were under antibiotic treatment, with Cotrimoxazole being the most implemented (30%).

### 3.2. Urine Cultures

Forty (49%) included urine samples (*n* = 85) were positive in urine culture. Monomicrobial cultures were obtained from 28 (70%) urine samples. The prevalence of uropathogens in monomicrobial cultures was *Escherichia coli* (89.2%), *Staphylococcus epidermidis* (3.5%), *Salmonella* spp. (3.5%), and *Citrobacter sedlakii* (3.5%). On the other hand, polymicrobial cultures were obtained from 12 (30%) of the analyzed urine samples, where *Escherichia coli* was found with other microorganisms in 9 (75%) samples. On MacConkey agar, three polymicrobial cultures with two different colonial morphologies (lactose positive and mucoid lactose positive) were observed, and both were identified as *E. coli*. The other microorganisms identified in polymicrobial cultures were *Buttiaxella agrestis*, *Moellerella wisconsensis*, *Citrobacter werkmanii,* and *Citrobacter gilenii*. Forty clinical isolates of *Escherichia coli* were obtained from all urine samples analyzed.

### 3.3. UPEC Morphotypes in Urine Sediment of Patients with UTI

**Figure 1:**
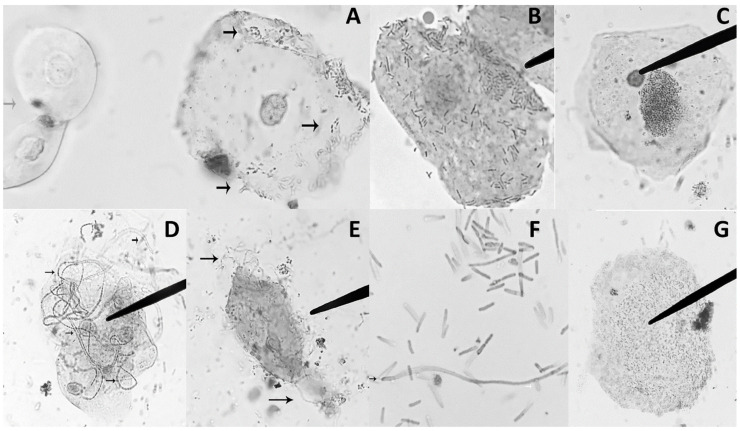
UPEC morphotypes observed in urinary sediment from patients with UTI. Light microscopic images of exfoliated uroepithelial cells and biofilm were stained with Sternheimmer-Malbin dye. (**A**) Right: Vesical epithelial cell with bacterial adherence (black arrows) and left: renal epithelial cell (gray arrow) (40×). (**B**) Vesical epithelial cell with bacterial adherence (100×). (**C**) Vesical epithelial cells and Intracellular bacterial communities (40×). (**D**) Vesical epithelial cells and filamentous *E. coli* (40×). (**E**) Vesical epithelial cell with cytolysis and filamentous *E. coli* (black arrows) (40×). (**F**) Filamentous *E. coli* (black arrow) (100×). (**G**) Biofilm.

UPEC morphotypes were observed in 24 (65%) of urine samples from patients with UTI caused by *E. coli* (*n* = 37). The most prevalent morphotypes were adherence (75%), IBC (54%), filamentous *E. coli* (25%), and biofilm (33%) (Figure 1). Morphotypes were frequently observed in combination (17/24 urine samples): Adherence+IBC (46%), Adherence+Biofilm (21%), Adherence+Filamentation (8%), Adherence+IBC+Filamentation (8%), and Adherence+IBC+Biofilm (8%). The etiologic agent of UTI in all urine samples with presence of morphotypes (*n* = 24) was *E. coli*; none of the urine samples with Gram positive bacteria showed evidence of morphotypes.

As expected, a higher prevalence of recurrent UTI episodes in patients with UPEC morphotypes in urinary sediments (71%) than in patients without them (46%) was observed; however, there was not statistical significance (*p* > 0.05). We also observed a higher prevalence of positive LE, and bacteriuria (>2+) in urine sediments with bacterial morphotypes than without it (Table 2b). Nevertheless, a significant difference was only found in the higher prevalence of bacteriuria (>2+) in urine samples with bacterial morphotype than without it (*p* > 0.05). It is important to note that urine cultures with a reduced number of CFU/mL were obtained more frequently from urine samples with bacterial morphotypes in comparison to those without (*p* < 0.0001) (Table 2b).

### 3.4. Prevalence of Virulence Associated Genes

**Figure 2:**
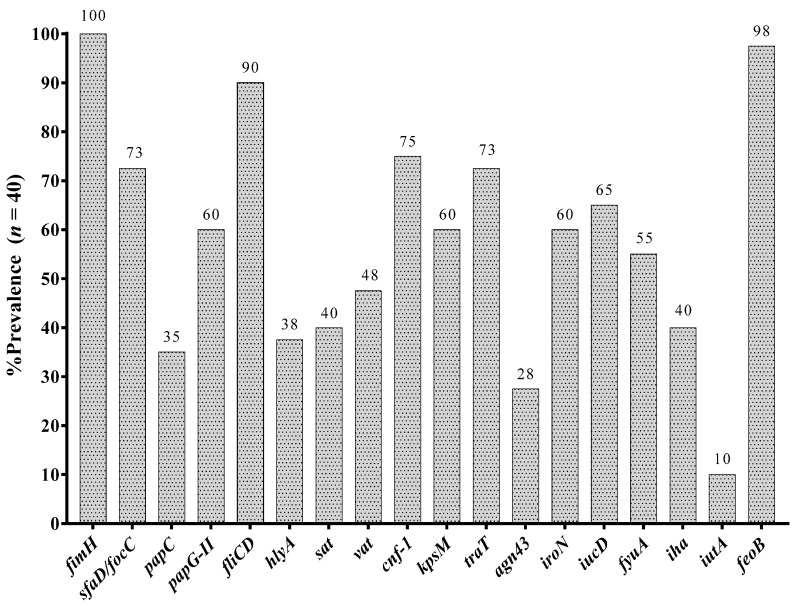
Prevalence of 18 virulence genes in analyzed clinical isolates of UPEC. ***fimH***: Fimbrial Adhesin of type 1 pilus; ***sfaD/focC***: S fimbriae minor subunit/F1C fimbriae chaperone; ***papC***: Type P pilus chaperone; ***papG-II***: Type P pilus Adhesin allele 2; ***fliCD***: Flagellin subunit/flagellar cap; ***hlyA***: α-hemolysin; ***sat***: Autotransporter secreted toxin; ***vat***: Vacuolating autotransporter toxin; ***cnf-1***: Necrotizing cytotoxic factor; ***kpsM***: Capsular variant; ***traT***: Serum resistance protein; ***agn43***: 43 antigen; ***iroN***: Salmochelin siderophore receptor; ***iucD***: Aerobactin; ***fyuA***: Yersiniabactin; ***iha***: Bifunctional enterobactin receptor/adhesin protein; ***iutA***: Ferric aerobactin receptor; ***feoB***: Ferrous iron transport protein B.

The most prevalent virulence associated genes were *fimH* (100%), followed by *feoB* (98%) and *fliCD* (90%). A high prevalence of the S fimbriae subunit/F1C fimbriae chaperone gene (*sfaD/focC*) (73%), P pilus Adhesin gene (*papG-II*) (60%), capsule associated gene *kpsM* (60%), and vacuolating autotransporter toxin gene *vat* (48%) was also observed (Figure 2). Three common virulence profiles were observed in twenty-three (58%) clinical isolates (Table 3) and are related to both lower and upper UTI.

We compared the prevalence of virulence-associated genes between clinical isolates obtained from urine samples in which morphotype was observed vs. clinical isolates obtained from urine samples without morphotype. Statistical significance was only observed for the higher prevalence of *hlyA* (62%, *p* = 0.04), and *vat* (77%, *p* = 0.01) genes in UPEC isolates with IBC in urinary sediment versus those without morphotype (26% and 33%, respectively).

On the other hand, we observed that *E. coli* clinical isolates analyzed showed co-occurrence of some of the virulence-associated genes that we determined, and statistical significance was observed in all cases (*p* < 0.05). Interestingly, these genes are within pathogenicity islands (PAI) of prototypes UPEC strains such as *E. coli* 536, *E. coli* CFT073, *E. coli* J96, and *E. coli* UMN026. Table 4 shows genes for which a positive correlation and statistical significance was observed.

### 3.5. Virulence Phenotypes of Clinical Isolates of UPEC

The prevalence of some of the most important virulence phenotypes of UPEC was investigated. We found that 98% of clinical isolates were motile, 70% were capsule producers, and only 5% produced the hemolysis phenotype. We tried to identify relationships between genotypes vs. these virulence phenotypes; however, only a statistically significant association between the higher prevalence of *kpsM* (*p* = 0.0367) and *iha* (*x*^2^ = 0.048) genes in capsule-producing isolates was observed.

**Figure 3:**
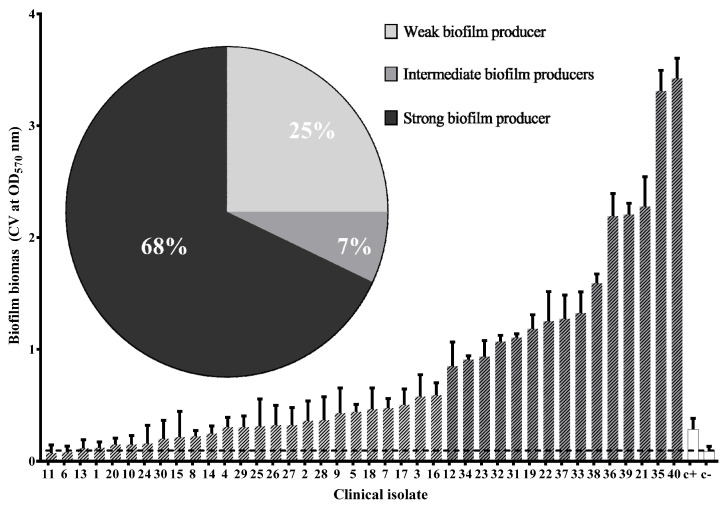
Biofilm production in clinical isolates of UPEC. The dotted line is at the level of the result obtained for the negative control. The biofilm biomass is expressed as the average OD at 570 nm of five independent experiments, error bars show standard deviation (SD). +C: Positive control (*Escherichia coli* 25922); -C: Negative control (sterile Mueller-Hinton Broth.). Dark bars show the stronger biofilm producers with the most statistical significance (*p* < 0.05) (Two-way ANOVA, Tukey’s multiple comparisons test), white bars show the implemented controls. Pie chart at the top shows the percentage of weak, intermediate, and strong biofilm forming UPEC clinical isolates.

All clinical isolates were biofilm producers (100%), but only 68% were strong biofilm producers (Figure 3). Comparing the prevalence of virulence genes in each biofilm producer group, a significant difference was observed only in the higher prevalence of *iucD* (*p* = 0.03) and *papC* (*p* = 0.05) genes in the strong biofilm producers.

**Figure 4:**
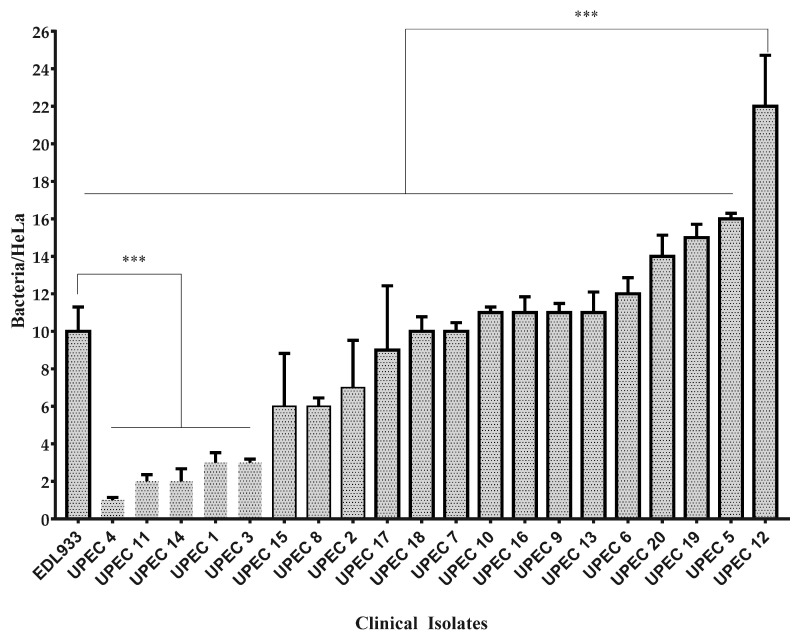
Adherence assay for UPEC clinical isolates. ***: *p* = 0.0001. One way ANOVA Tukey’s multiple comparisons test. Adherence groups are shown in the graph. Bars with thick line: strongly adherent group; bars with thin line: moderately adherent group; bars without line: low adherent group. Total HeLa cells and adherent bacteria were counted in 10 fields at 40X objective. The results are expressed as the average number of adherent bacteria from three independent experiments. Error bars show standard error of the mean (SEM).

For adherence assay, twenty clinical isolates were selected according to their virulence profile and phylogenetic groups. Twenty-five percent of selected isolates were low adherent to HeLa cells, 15% were classified as moderately adherent, and 60% were strongly adherent. Fifteen (75%) of selected UPEC were more adherent than positive control (*E. coli* EDL 933) (*p* = 0.0001) (Figure 4). Interestingly, UPEC 12 was the most adherent and virulent isolate with 16/18 virulence associated genes.

Positive correlation between strongly adherent UPEC with a higher prevalence of *papC* gene (r = 0.471, *p* = 0.036) was observed. No statistical significance between adherence groups, biofilm formation groups, phylogenetic groups, and UPEC morphotypes in urine was found (*p* > 0.05).

### 3.6. Antibiotic Resistance Phenotypes

**Figure 5:**
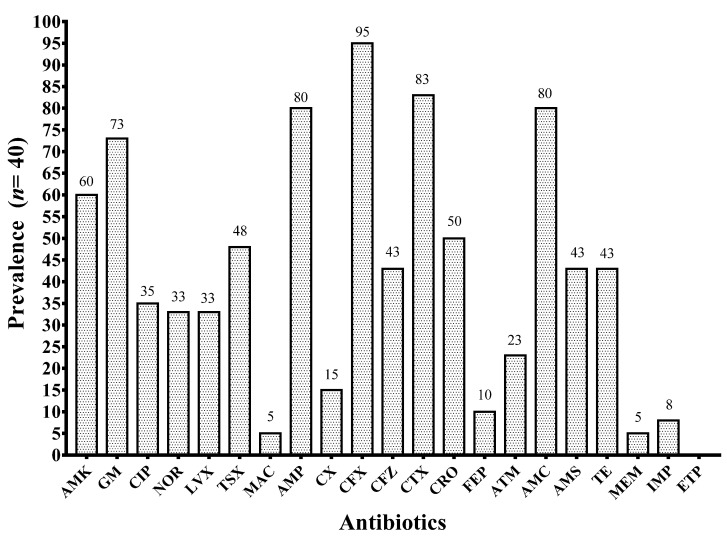
Antibiotic resistance prevalence. **AMK**: Amikacin; **GM**: Gentamicin; **CIP**: Ciprofloxacin; **NOR**: Norfloxacin; **LVX**: Levofloxacin; **TSX**: Cotrimoxazole; **MAC**: Nitrofurantoin; **AMP**: Ampicillin; **CX**: Cefoxitin; **CFX**: Cefuroxime; **CFZ**: Ceftazidime; **CTX**: Cefotaxime; **CRO**: Ceftriaxone; **FEP**: Cefepime; **ATM**: Aztreonam; **AMC**: Amoxicillin/Clavulanate; **AMS**: Ampicillin/Sulbactam; **TE**: Tetracyclin; **MEM**: Meropenem; **IMP**: Imipenem; **ETP**: Ertapenem.

Obtained clinical isolates showed a higher resistance to antibiotics of the β-lactam family, mainly ampicillin (80%), second and third generation cephalosporins: Cefuroxime (95%), Cefotaxime (83%), and the inhibitor combined β-lactamic: Amoxicillin/Clavulanate (80%). High resistance was also observed for aminoglycosides: Amikacin (60%) and Gentamicin (73%) (Figure 5). On the other hand, the isolates were predominantly sensitive to nitrofurantoin, one of the most widely used antibiotics in the treatment of UTI. According to Magyorakos criteria, only 7.5% of clinical isolates were not multidrug resistant (NMDR), while 85% were multidrug resistant (MDR), and 7.5% were classified as extremely resistant (XDR). Additionally, one clinical isolate was sensitive for all tested antibiotics. Distribution of antibiotic resistant in all clinical isolates is shown in Supplementary Material Table S2. Most common resistance profile was AMK, GM, AMP, CFX, CTX, and AMC, found in 17 (43%) clinical isolates. No statistical significance was observed between biofilm producers groups, bacterial morphotypes, and antibiotic resistance [26].

### 3.7. Phylogenetic Groups of Obtained Clinical Isolates

The prevalent phylogenetic group of the bacterial population studied was B2 (27.5%), followed by B1 (22.5%), E (15%), and C (10%). Twenty-five percent of the clinical isolates belonged to unknown phylogenetic groups (NT). No isolates belonging to groups A, D, and F were observed.

**Figure 6:**
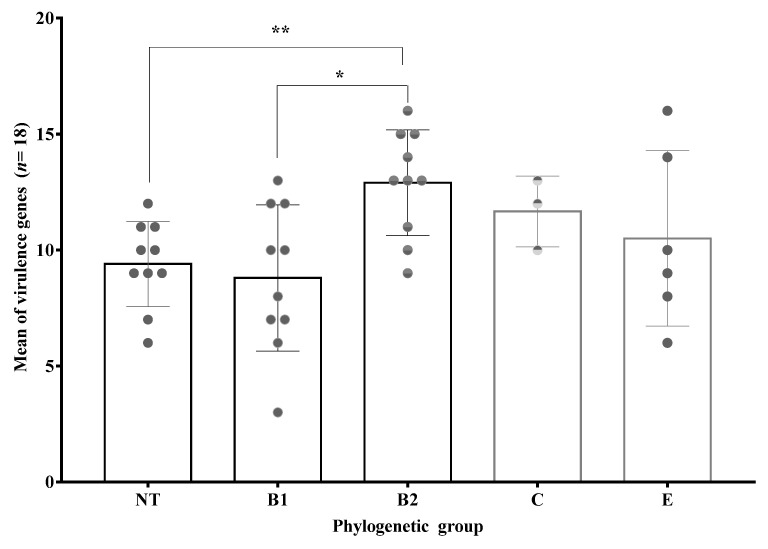
Mean of virulence by phylogenetic groups. **: *p* = 0.01; *: *p* = 0.04. One way ANOVA Tukey’s multiple comparisons test. Error bars show standard deviation (SD) and each gray dot represent clinical isolates of specific phylogenetic group.

According to the mean of virulence genes in each phylogenetic group, more virulent UPEC isolates belong to B2 phylogenetic group. However, statistical significance was observed only in the higher mean of virulence of B2 vs. B1 and NT phylogenetic groups (Figure 6). We also observed that the prevalence of specific virulence genes was different between phylogenetic groups. In this sense, *papC*, *iroN*, and *fyuA*, which are genes related to highly pathogenic *E. coli*, were most prevalent in isolates from B2 phylogenetic group (Table 5).

Analyzing results of virulence phenotypes, we observed a positive correlation between phylogenetic group B2 and hemolysis (*r* = 0.37, *p* = 0.017) and a negative correlation between phylogroup NT and capsule production (*r* = −0.38, *p* = 0.016). In adherence assays, a negative correlation between high adherent bacteria and phylogenetic group C was observed. No statistically significant association between biofilm producers’ groups, morphotypes in urine sediment, and phylogenetic group was found.

**Figure 7:**
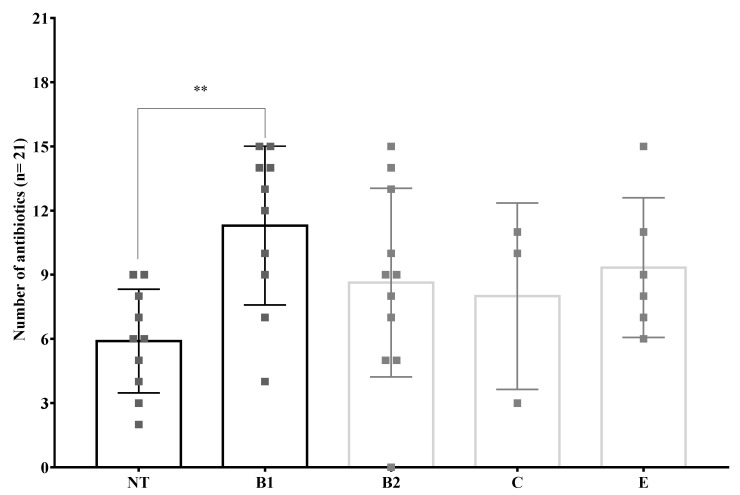
Mean of antibiotic resistance by phylogenetic groups. **: *p* = 0.01. One way ANOVA Tukey’s multiple comparisons test. Error bars show standard deviation (SD) and each gray square represent clinical isolates of specific phylogenetic group.

Regarding antibiotic resistance, a statistically significant difference was only observed in the higher number of antibiotics to which clinical isolates of phylogenetic group B1 showed resistance compared to those belonging to phylogenetic group NT (Figure 7).

## 4. Discussion

In this study, the prevalence of bacterial morphotypes in urine sediments, virulence associated genes, virulence phenotypes, and phylogenetic groups of UPEC were investigated.

In polymicrobial cultures, together with *Escherichia coli*, we found some atypical urinary tract pathogens. These were *Moellerella wisconsensis* and *Buttiaxella agrestis*, which are microorganisms commonly reported in infectious processes in other animal species, such as dogs or cats, and only in a few reports as causative agents of human infections after surgical procedures or in immunosuppressed patients [27,28,29]. In this sense, it is important to mention that in this work both isolates were obtained from diabetic patients that expressed having at least one previous UTI episode. It is reported that diabetic patients have four times more probability of developing infectious diseases, including UTI, and the etiology of these infections include atypical pathogens, this being a possible explication for our results [30,31,32]. Interestingly, to our knowledge, this is the first report of *Butiaxella agrestis* and *Moellerella wisconsensis* isolated from UTI in México.

Nowadays polymicrobial infections have gained importance due to their probable association with therapeutic failures and horizontal gene transfer between pathogens [33,34]. In this sense, in polymicrobial cultures, we observed the presence of some species of the genus *Citrobacter*, such as *Citrobacter sedlakii*, *Citrobacter gillenii*, and *Citrobacter werckmanii*. These microorganisms are considered emerging pathogens in several infectious processes including urinary tract infections [35,36], and are also resistant to antibiotics used as treatment of UTI, mainly Cotrimoxazole, quinolones, and β-lactam antibiotics [27,28,29,36,37,38,39]. Therefore, it would be interesting to investigate and compare the resistance characteristics of these microorganisms with those of *E. coli* obtained from the same urine sample.

The presence of bacterial morphotypes in urine is important due to their association with immune evasion and antibiotic resistance. In this regard, we found that 65% of the *E. coli* isolates were obtained from urines samples with morphotypes. These results are higher than those reported by Robino et al. in 2013 and 2014 [10,40], who found morphotypes in only 22.6% of the analyzed urine samples. Interestingly, 17 (71%) of the urine samples that showed morphotypes were negative in urine culture. However, by applying vortex to release the intracellular bacteria, the CFU/mL counts increased, and the urine cultures were positives (>100,000 CFU/mL). Therefore, when IBC are observed, we suggest the implementation of bacterial releasing methods to reduce false negatives in urine cultures. On the other hand, in seven of the urine samples with morphotype, positive urine cultures were obtained despite the presence of IBC. This could be explained by the number of extracellular bacteria present in the sample, because part of the process of maturation of the IBC leads to the release of bacteria into the extracellular medium.

We also observed that bacterial morphotypes were more frequent in urine sediments from patients with recurrent UTI episodes than patients without it. This is in accordance with the reported by Robino, Rosen, and Martinez-Figueroa [11,17,40]. These results suggest that the method used in the clinical diagnosis of UTI needs to be modified, the search for these UPEC morphotypes in urinary sediment must be done routinely to avoid misdiagnosis, and Sternheimer-Malbin dye could be implemented for detection of these bacterial morphotypes. In addition to IBC and filamentous bacteria in urinary sediment, we considered it important to report the presence of bacterial adherence to bladder cells and biofilms in urinary sediment, since both play a significant role in the pathogenic mechanism of UPEC and could be involved in the persistence of this pathogen in the urinary tract. To our knowledge, this is the first report of prevalence of UPEC bacterial morphotypes in urinary sediment of Mexican population.

When analyzing the clinical data collected from the patients, we found that 30% of the women with recurrent UTI episodes were undergoing or had completed treatment with Cotrimoxazole. In Mexico, there are several reports demonstrating the high resistance of clinical isolates of UPEC to this antimicrobial agent which has led to considering its therapeutic efficacy. However, it would be important to continue with research focused on the determination of local susceptibility profiles for the antibiotics included in the basic treatment for UTI in Mexico, since it is known that resistance profiles can differ depending on the geographic area [14,16,41,42,43,44,45].

Antimicrobial resistance is currently a challenge in health because therapeutic options are reduced. In this study we found that the clinical isolates obtained were predominantly multidrug resistant (93%) and showed a high resistance to antibiotics implemented in the basic treatment of UTI, mainly aminoglycosides, β-lactams, and cotrimoxazole. These results are in agreement with those reported in previous studies [14,16] in Mexico, which highlights the urgent need to search for therapeutic alternatives for the treatment of UTI.

Regarding virulence, we observed that all clinical isolates carried the *fimH* gene. Our results are similar to those reported in previous works in Peru and Ethiopia, where a prevalence of 98% and 82% for *fimH* in clinical isolates of UPEC was reported [46,47]. In Mexico, Miranda-Estrada et al. 2017; Morales-Espinosa et al. 2016, López-Banda. 2014, and Ballesteros-Monrreal et al. 2020 reported a prevalence of 96%, 100%, 86%, and 100%, respectively, for this Adhesin [14,15,48,49]. This was not unexpected since *fimH* is crucial in the development of UPEC uropathogenic mechanism, including IBC formation.

Among the identified genes associated with pyelonephritis, the gene associated with flagellum (*fliCD*) was the most prevalent (90%). This prevalence is higher than the reported in previous studies conducted by Tabasi et al., 2016 in Iran and Qingqing et al., 2017 in China where a prevalence of 68% and 15%, respectively, was observed [50,51]. In this regard, in Mexico there is scarce evidence concerning the prevalence of this gene in clinical isolates of UPEC; however, in recent reports by Ordaz-López in Mexico City and Ballesteros-Monrreal in the state of Puebla, the *fliC* gene has been observed in 25% and 30%, respectively [14,52]. On the other hand, the *papG-II* gene which codify for type P pilus Adhesin was also highly prevalent (60%). These results are different to those reported in other work in Mexico by Bravata-Alcantara and Luna-Pineda, who reported a prevalence of 21.5% and 15.4%, respectively [53,54]. Our results suggest that clinical isolates from the State of Sonora have a greater potential to cause upper UTIs compared to other Mexican states. Additionally, despite the scarce existing information, the reports available in Mexico show a higher prevalence of these genes in our country compared to others [55,56].

Similarly, a high prevalence of the *sfaD/focC* gene was also observed (73%), which is associated with both pili S and pili F1C. This gene is of interest because it is not only associated with pyelonephritis, but also meningitis and septicemia in adults [57]. Additionally, we observed that 72.5% of the clinical isolates that presented the *sfaD/focC* gene also presented the *papG-II* gene, associated with type P pili. This could be explained by the fact that both genes are harbored within the pathogenicity island (PAI) III of *Escherichia coli* 536 [58]. Interestingly, in addition to *sfaD/focC* and *papG-II* a high prevalence of clinical isolates with co-occurrence of virulence genes reported in PAI was observed (Table 4). Presence of PAIs could indicate a high pathogenic potential, so it would be interesting to determine in the future the presence of these genetic elements in our clinical isolates.

We also observed a considerable prevalence of the genes *kpsM* (60%), *sat* (40%), and *hlyA* (38%). These results are similar to those previously reported in Mexico [15,57,59]. The *kpsM* gene is associated with capsule production, and it is known that capsules may contribute to immune evasion, mainly in serum resistance, phagocytosis, and resistance to death by neutrophils and monocytes [7,60]. On the other hand, the *sat* and *hlyA* are toxigenicity associated genes involved mainly in upper UTI. The Sat protein has been reported as a vacuolating cytotoxin in cultured mammalian bladder and kidney cells [61]. While HlyA protein is a toxin with cytolytic effect, it is also involved in iron acquisition, since iron can be released from damaged cells [62], which is subsequently captured by siderophores produced by UPEC. Additionally, this protein can act as an immunomodulator at sublytic concentrations favoring UPEC immune evasion, even during bacteriemia [9,63,64]. Despite the high prevalence of the *hlyA* gene, we only observed hemolysis phenotype in 5% of the analyzed clinical isolates. These results are similar to those previously reported by our work group, where a prevalence of the gene in clinical isolates from Sonora of 38–56% and a coincidence with the hemolysis phenotype of 12–16% were observed [14]. The higher prevalence of the *hlyA* gene compared to its respective phenotype could be explained by the fact that the HlyA protein is the immature toxin, which requires a prior acetylation step to generate its lytic effect.

The most common phenotype observed was biofilm production (100%); 60% of the clinical isolates were strong biofilm producers. This phenotype is important since it is associated with antimicrobial resistance. When analyzing the prevalence of each of the virulence-associated genes among biofilm-producing groups, we observed a higher prevalence of the *iucD* and *papC* genes in the strong biofilm producers. Interestingly, neither of the aforementioned genes are directly implicated in biofilm formation, but both have been reported to be associated with genomic islands. Similar results were found in adherence phenotypes, where a positive correlation was found between the *papC* gene and the strong adherence group; this gene was not observed in any of the isolates that presented weak or medium adherence. *papC* encodes for the helper chaperone protein of the pyelonephritis-associated pili and is not directly associated with the adherence phenotype, so, as with biofilm production, it is probably that another gene associated with the same pathogenicity island in which *papC* or *iucD* are harbored is directly involved with these phenotypes.

The prevalence of phylogenetic groups in the obtained isolates was also examined. Interestingly, a high prevalence of clinical isolates that could not be phylogenetically classified (25%) was observed, which could indicate the presence of new phylogenetic groups in Sonora, and these results are in accordance with those previously reported by our work group in Puebla and Sonora [14].

It is thought that the most pathogenic isolates are clustered in phylogenetic groups B2 and D, while the most resistant and commensal isolates are located in groups A and B1. Comparing the average of virulence associated genes by phylogenetic group, we observed statistically significant differences between the highest number of virulence genes in phylogroup B2 compared to phylogroup B1 and non-typeable (NT), but not with phylogroups C and E. However, no statistically significant difference was found between the average number of virulence genes present in phylogroups B1, NT, C, and E. Similar results were observed in antibiotic resistant. Clinical isolates classified as B1 were more resistant than NT (*p* < 0.05), but no than the phylogroups B2, C, and E. This could suggest that virulence and antibiotic resistance are not restricted to the specifics phylogenetics groups.

In summary, we observed that all clinical isolates presented the *fimH* gene, which is indicative of pathogens that have the capacity to cause lower urinary tract infections. In addition, 65% of the UPEC with the *fimH* gene presented bacterial morphotypes in urinary sediment, indicating that they are bacteria with the ability to cause lower UTI and internalize, forming IBC or bacterial filaments, which allow them to avoid the host immune response, resist the effects of antimicrobial treatments, and persist in the urinary tract leading to recurrent episodes of UTI. Seventy-three percent of clinical isolates also present the *fliCD* gene or the motile phenotype and any of the *papG-II*, *papC,* or *sfaD/focC* genes that are related to renal adherence; therefore, these pathogens have the capacity to cause both lower UTI and upper UTI. Finally, a high percent of the obtained isolates presented each of the characteristics described above together with the α-hemolysin genes or the secreted autotransporter toxin (*sat*) as well as the gene *kpsM* or capsule phenotype, suggesting highly pathogenic UPEC which are potentially capable of causing both types of UTI, evading the host immune system, resisting antibiotic treatment, persisting in the urinary tract, and causing recurrent UTI. Additionally, these bacteria have potential to induce renal damage, and gain access to the bloodstream and cause bacteremia.

## 5. Conclusions

In conclusion, UPEC’s clinical isolates obtained from adult women in Sonora were MDR and had a high pathogenic potential to cause lower and upper UTI. In Mexico, the actual prevalence of UPEC bacterial morphotypes in urinary sediment is unknown. However, the available evidence indicates that it is a common phenomenon in the Mexican population and is associated not only with recurrence of UTI but also with false negatives in urine culture, which considerably delays the treatment of the infectious process and could lead to more serious complications. Therefore, diagnostic methods in the clinical laboratory should include the search for these morphotypes in urinalysis.

## Figures and Tables

**Table 1:** Primers used for the detection of virulence associated genes and molecular identification of clinical isolates of *E. coli*.

Gene	Sequence (5′-3′)	Size Product	Tm °C	Reference
*fimH*	Forward: TTATGGCGGCGTGTTATC Reverse: TCCCTACTGCTCCTAACG	545 bp	54	This study
*sfaD/focC*	Forward: AGGCAAATGGACAGGTATGG Reverse: TCACCCAGAACAAACTTTCC	412 bp		This study
*papG-II*	Forward: ATTCACCATAGAGGCGACTG Reverse: ATCATTATGCGGCTCAGAC	237 bp		This study
*papC*	Forward: TTCTCTCTCCCTCAATACGG Reverse: TTATAACCTCAACGGGACGG	926 bp		This study
*fliCD*	Forward: CCGAATCAGAGTTAGTTCCG Reverse: CCCAGCGATGAAATACTTGC	610 bp	60	This study
*sat*	Forward: GTTGGCAAACAGGTCAAAC Reverse: CTCGGAGTATTGGCTTCAG	809 bp		This study
*hlyA*	Forward: GATACGCTGATAGGTGAG Reverse: CCAGGTGTGACTCAATAC	564 bp	58	This study
*kpsM*	Forward: CCAGAGTAGATATGACCAG Reverse: CTACGAGAAATACGAACAC	409 bp		This study
*agn43*	Forward: CACACAGCCACTAATACC Reverse: CACCTGAATACCCTTACC	488 bp	58	This study
*vat*	Forward: ATACAGTCTCGTCTCTGG Reverse GTGACAGTCCCTTTATCC	670 bp		This study
*cnf-1*	Forward: CAGACTCATCTTCACTCG Reverse: AGACAGAGACCTTACGAC	551 bp		This study
*traT*	Forward: TGGTATAGTTCACATCTTCC Reverse: TAAAGCCTACTACTGGATTC	233 bp		This study
*fyuA*	Forward: CGCCAGTAAACAATCTTCCC Reverse: CCCAAACACCATATCAACGG	937 bp	60	This study
*iucD*	Forward: CGTGAGACCCAGTTTATTTCC Reverse: GGGCTGCTGAAGATATGAATAACC	334 bp		This study
*iroN*	Forward: CAGAATGATGCGGTAACTCC Reverse: CGTGAGACCCAGTTTATTTCC	435 bp		This study
*iutA*	Forward: GTTCACGCTCTTTGTCAGG Reverse: GGGCTTAATCTCGGGAAAGG	801 bp		This study
*feoB*	Forward: GTCTAACCTTGAGCGTAACC Reverse: GGCGAGGAAGATAGTCAGC	736 bp		This study
*iha*	Forward: TGTGCTCTGGTTTGATATGG Reverse: CATTCTGGGTGCCTTATATCC	594 bp		This study
*ybbW*	Forward: TGATTGGCAAAATCTGGCCG Reverse: ATACTGGCAATCAGTACGCC	667 bp		[20]

***fimH***: Type 1 pilus Adhesin; ***sfaD/focC***: S and Dra fimbriae; ***papC***: Type P pilus chaperone; ***papG-II***: Type P pilus Adhesin allele 2; ***fliCD***: Flagellin subunit/flagellar cap; ***hlyA***: α-hemolysin; ***kpsM***: Capsular variant; ***sat***: Autotransporter secreted toxin; ***agn43***: Antigen 43; ***vat***: Vacuolating autotransporter toxin; ***cnf-1***: Necrotizing cytotoxic factor; ***traT***: Complement resistance associated protein; ***fyuA***: Ferric yersiniabactin uptake receptor; ***iucD***: Aerobactin; ***iroN***: Salmocheline receptor; ***iutA***: Aerobactin receptor; ***feoB***: Ferrous iron transporter; ***iha***: IrgA homologue Adhesin/enterobactin receptor; ***ybbW***: Allantoin receptor.

**Table 2:** Urinalysis, comorbidities, and urine culture results.

(a) Included Patients’ Groups (*n* = 85)				(b) Patients with UTI Caused by UPEC (*n* = 37)		
	With UTI *n* = 40 (%)	Without UTI *n* = 45 (%)	*p*	With Morphotype *n* = 24 (%)	Without Morphotype *n* = 13 (%)	*p*
Urinalysis						
pH:						
5.0–6.5	33 (83)	39 (87)	0.55	22 (92)	11 (85)	0.6
7.0–8.0	7 (17)	6 (13)		2 (8)	2 (15)	
LE:						
Positive	25 (63) *	12 (27)	0.002	16 (67)	7 (53)	0.49
Negative	15 (37)	33 (73)		8 (33)	6 (46)	
Pyuria:						
Positive (>5 WBCs/HPF)	31 (78) *	13 (29)	<0.0001	14 (58)	10 (85)	0.72
Negative (<5 WBCs/HPF)	9 (22)	32 (71)		10 (42)	4 (15)	
Bacteriuria:						
>2+	23 (58)	0 (0)	<0.0001	7 (29)	13 (100)	<0.0001
<2+	17 (42)	45 (100) *		17 (71) *	0 (0)	
**Comorbidities**						
Diabetes	11 (28)	12 (27)	>0.99	7 (29)	4 (31)	>0.99
Renal failure	3 (8)	3 (7)	>0.99	1 (4)	2 (15)	0.28
Hypertension	10 (25)	12 (27)	>0.99	5 (20)	3 (23)	>0.99
Diagnosed ITU	6 (15)	2 (4)	0.12	3 (13)	3 (23)	0.64
Vaginal infection	4 (10)	4 (9)	>0.99	0 (0) *	3 (23)	0.037
Pregnancy	3 (8)	1 (2)	0.33	2 (8)	1 (8)	>0.99
Hypothyroidism	1 (3)	5 (11)	0.2	0 (0)	1 (8)	0.35
**Urine culture**						
<100,000 CFU/mL	17 (43)	45 (100) *	<0.0001	17 (71)	-	<0.0001
>100,000 CFU/mL	20 (50)	0 (0)		7 (29)	13 (100%)	

**LE**: Leucocyte esterase; **WBCs/HPF**: White blood cells per high power field. The higher prevalence of LE, pyuria, and bacteriuria in patients with UTI was significant, in comparison to the same prevalence in patients without UTI (*p* < 0.05). The higher prevalence of bacteriuria <2+ in urine sediments with bacterial morphotype than without it was significant (*p* < 0.05). *p* value was determined by Fisher’s exact test. Values with statistical significance are indicated with *.

**Table 3:** Common virulence profile in analyzed clinical isolates of UPEC.

Virulence Profile	Clinical Isolates
*fimH, feoB, fliCD, cnf-1, sfaD/focC*	2,4–6,9–13,16,20,22–24,29,31,33–37,39–40
*fimH, feoB, fliCD, cnf-1, sfaD/focC, traT, papG-II, kpsM*	2,5,6,9,12,13,20,23,37
*fimH. feoB, fliCD, cnf-1, sfaD/focC, traT, papG-II, kpsM, vat, sat*	5,6,12,37

**Table 4:** Co-occurrence of virulence genes related with PAI in clinical isolates of UPEC.

Gene	% (*n* = 40)	*r*	*p*	Reported PAI ^a^
***papG-II***:				PAI I, IICFT073, PAI I-V536, PAI I-IIJ96
*papC*	32.5	0.49	0.001	PAI I-IICFT073
*vat*	40	0.47	0.002	Not named PAI Ec222
*iroN*	45	0.38	0.017	PAI III536
*fyuA*	42.5	0.39	0.012	PAI IICFT073, PAI III, IV536
*sfaD/focC*	72.5	0.54	<0.001	PAI I, IICFT073, PAI I-IVJ96, PAI I-IIJ96, PAI I-III536
***papC***:				PAI IICFT073
*iroN*	30	0.38	0.0141	PAI III536
*fyuA*	30	0.45	0.003	PAI IICFT073, PAI III, IV536
***sat***:				PAI IICFT073
*vat*	27.5	0.35	0.028	Not named PAI Ec222
*iroN*	35	0.46	0.002	PAI III536
*fyuA*	35	0.53	<0.001	PAI IICFT073, PAI III, IV536
*kpsM*	32.5	0.35	0.02	PAI V536
*iucD*	35	0.39	0.01	PAI IICFT073, PAI III-IV536, Not named PAI UMN026
***hlyA***:				PAI ICFT073, PAI I-II536, PAI I-IIJ96
*kpsM*	35	0.53	<0.001	PAI V536
*cnf-1*	35	0.33	0.04	PAI IIJ96
***vat***:				Not named PAI Ec222
*cnf-1*	42.5	0.32	0.05	PAI IIJ96
***cnf-1***:				PAI IIJ96
*iha*	37.5	0.35	0.025	PAI I, IICFT073, PAI I-IVJ96, PAI I-IIJ96, PAI I-II536
*KpsM*	52.5	0.35	0.025	PAI V536
***iroN***:				PAI III536
*fyuA*	47.5	0.59	<0.001	PAI IICFT073, PAI III, IV536

Statistical significance and correlation value (*r*) was obtained with Pearson correlation coefficient. For Pearson correlation test, we statistically analyzed all genotypes, phenotypes, and phylogenetic groups against another; *r* values were obtained and *p* value was confirmed with Fisher’s exact test or Chi-square. Correlation is significant at 0.01 level (2-tailed). ^a^: Accession link to Pathogenicity Island Database (PAI DB) *E. coli* Pathogenicity Island http://www.paidb.re.kr/browse_pais.php?m=p&SPC=Escherichia%20coli (accessed on 26 October 2021).

**Table 5:** Virulence genes distribution between phylogenetic groups of UPEC clinical isolates.

Gene	NT *n* = 10 (%)	*p*	B1 *n* = 10 (%)	*p*	B2 *n* = 11 (%)	*p*	C *n* = 3 (%)	*p*	E *n* = 6 (%)	*p*	Total *n* = 40 (%)
*fimH*	10 (100)	1	10 (100)	1	11 (100)	1	3 (100)	1	6 (100)	1	14 (35)
*papC*	2 (20)	0.7	2 (20)	0.7	7 (64)	0.02	0 (0)	0.53	3 (50)	0.64	40 (100)
*papG-II*	6 (60)	1	4 (40)	0.15	9 (82)	0.13	3 (100)	0.26	2 (33)	0.19	29 (73)
*sfaD/focC*	8 (80)	0.69	9 (90)	0.23	7 (64)	0.45	3 (100)	0.54	2 (33)	0.03	24 (60)
*fliCD*	10 (100)	0.55	9 (90)	1	10 (91)	1	3 (100)	1	4 (67)	0.09	36 (90)
*cnf-1*	8 (80)	1	7 (70)	0.68	9 (82)	0.69	2 (67)	1	4 (67)	0.62	16 (40)
*vat*	4 (40)	0.72	4 (40)	0.72	6 (55)	0.72	2 (67)	0.59	3 (50)	1	15 (38)
*sat*	3 (30)	0.71	1 (10)	0.06	7 (64)	0.08	2 (67)	0.55	3 (50)	0.66	24 (60)
*hlyA*	4 (40)	1	4 (40)	1	6 (55)	0.27	0 (0)	0.27	1 (17)	0.38	19 (48)
*feoB*	10 (100)	1	9 (90)	1	11 (100)	1	3 (100)	1	6 (100)	1	30 (75)
*iucD*	4 (40)	0.12	5 (50)	0.27	9 (82)	0.26	2 (67)	1	6 (100)	0.07	29 (73)
*iroN*	4 (40)	0.15	3 (30)	0.05	10 (91)	0.02	3 (100)	0.26	4 (67)	1	11 (28)
*fyuA*	4 (40)	0.3	1 (10)	0.002	11 (100)	0.0003	2 (67)	0.99	4 (67)	0.67	24 (60)
*iha*	1 (10)	0.03	4 (40)	1	6 (55)	0.29	2 (67)	0.55	3 (50)	0.66	26 (65)
*iutA*	0 (0)	0.55	3 (30)	0.71	9 (82)	1	0 (0)	1	0 (0)	1	22 (55)
*traT*	9 (90)	0.23	4 (40)	0.01	8 (73)	1	2 (67)	1	6 (100)	0.16	16 (40)
*kpsM*	5 (50)	0.48	5 (50)	0.48	9 (82)	0.14	1 (33)	0.55	4 (67)	0.63	4 (10)
*agn43*	2 (20)	0.69	4 (40)	0.41	1 (9)	0.23	2 (67)	0.17	2 (33)	1	39 (98)

The *p* values were calculated with Fisher Exact Test comparing the prevalence of the virulence gene in each phylogenetic group with all other combined groups. Values significantly higher than the other groups are showed in red boxes, while values significantly lower than the other groups are shown in purple boxes.

## Supplementary Materials

The following are available online at https://www.mdpi.com/article/10.3390/microorganisms9112381/s1, Table S1: Genes and phenotype traits from the strains selected for adherence assays. Table S2: Antibiotic resistance distribution in obtained clinical isolates of UPEC.
