# Supplementary material for: Bacterial Morphotypes as Important Trait for Uropathogenic E. coli Diagnostic; a Virulence-Phenotype-Phylogeny Study
Source: Microorganisms. 2021 Nov 18;9(11):2381. doi: 10.3390/microorganisms9112381 (PMC8621242; doi:10.3390/microorganisms9112381)
Supplement: Supplementary file 1 [file microorganisms-09-02381-s001.zip › Supplementary Material File S1.pdf]

Supplementary Table S1. Genes and phenotype traits from the strains selected for adherence assays.

Isolate	Genotype									Phenotype		
	fimH	*fliCD*	*sfaD/focC*	*papG-II*	*kpsM*	*iha*	*papC*	*agn43*	Phylogroup	Biofilm	Capsule	Morphotype
1	+	+	neg	+	+	+	neg	neg	B2	neg	+	+
2	+	+	+	+	+	neg	neg	neg	B1	+	neg	+
3	+	+	+	+	neg	+	neg	+	C	+	neg	+
4	+	+	+	+	neg	+	neg	+	C	neg	+	+
5	+	+	+	+	+	neg	neg	neg	C	neg	+	+
6	+	+	+	+	+	+	neg	+	B2	neg	+	neg
7	+	neg	neg	neg	neg	neg	neg	+	B1	neg	+	neg
8	+	+	+	neg	+	neg	neg	neg	ND	neg	neg	+
9	+	+	+	+	+	neg	+	neg	B2	+	+	neg
10	+	+	+	+	neg	neg	+	neg	ND	neg	neg	neg
11	+	+	+	neg	+	neg	neg	+	ND	neg	neg	+
12	+	+	+	+	+	+	+	+	E	+	+	neg
13	+	+	+	+	+	neg	neg	+	ND	neg	+	+
14	+	neg	neg	neg	+	neg	neg	neg	E	neg	+	neg
15	+	+	neg	neg	neg	+	neg	+	E	+	+	+
16	+	+	+	neg	+	+	neg	+	B1	neg	+	+
17	+	+	neg	+	neg	+	+	neg	ND	+	neg	+
18	+	+	+	+	neg	neg	+	neg	B2	+	+	+
19	+	+	neg	+	neg	neg	neg	neg	ND	+	neg	+
20	+	+	+	+	+	neg	neg	neg	ND	neg	+	+

Symbols denotes a PCR positive reaction or a positive phenotype in each strain, “neg” denotes a negative reaction or a negative phenotype in each strain.
