# Supplementary material for: Bacterial Morphotypes as Important Trait for Uropathogenic E. coli Diagnostic; a Virulence-Phenotype-Phylogeny Study
Source: Microorganisms. 2021 Nov 18;9(11):2381. doi: 10.3390/microorganisms9112381 (PMC8621242; doi:10.3390/microorganisms9112381)
Supplement: Supplementary file 1 [file microorganisms-09-02381-s001.zip › Supplementary Material File S2.pdf]

Supplementary Table S2. Antibiotic resistance distribution in obtained clinical isolates of UPEC

Isolate	Amgs			Fqns		Sphs	Nts	Pncn	2nd Cps		3rd Cps			4th Cps	Mnbm	β-lactams + Inh		Ttyn	Cpnm			Classification
	AMK	GM	CIP	NOR	LVX	TSX	MAC	AMP	CX	CFX	CFZ	CTX	CRO	FEP	ATM	AMC	AMS	TE	MEM	IMP	ETP	
1	1	1	1	1	1	0	0	1	0	1	0	1	0	0	0	1	1	0	0	0	0	MDR
2	0	1	1	1	1	1	0	1	0	1	1	1	1	1	1	1	1	1	0	0	0	XDR
3	1	1	0	0	0	1	0	1	0	1	0	1	1	0	0	1	1	1	0	0	0	MDR
4	1	1	0	0	0	0	0	0	0	1	0	0	0	0	0	0	0	0	0	0	0	NMDR
5	0	0	0	0	0	1	0	1	1	1	1	1	1	1	1	1	1	0	0	0	0	MDR
6	0	1	1	1	1	1	0	1	0	1	0	0	0	0	0	1	0	1	0	0	0	MDR
7	1	1	0	0	0	0	0	1	0	1	0	0	0	0	0	1	0	1	1	0	0	MDR
8	0	0	0	0	0	0	0	0	0	1	0	0	0	0	0	0	0	1	0	0	0	NMDR
9	0	0	0	0	0	0	0	0	0	0	0	0	0	0	0	0	0	0	0	0	0	NMDR
10	0	1	0	0	0	0	0	1	0	1	1	1	0	0	1	0	0	1	0	0	0	MDR
11	0	1	1	1	1	0	0	1	0	1	0	1	0	0	0	1	0	0	0	0	0	MDR
12	1	1	0	0	0	0	0	1	0	1	0	1	0	0	1	1	1	1	0	0	0	MDR
13	0	0	0	0	0	0	0	0	0	1	0	1	0	0	0	0	0	0	0	0	0	MDR
14	1	0	1	0	0	0	0	0	0	1	0	1	1	0	0	0	0	1	0	0	0	MDR
15	0	1	0	0	0	1	0	1	0	1	0	1	0	0	0	1	0	1	0	0	0	MDR
16	0	1	0	0	0	0	0	0	0	1	0	1	0	0	0	1	0	0	0	0	0	MDR
17	1	1	0	0	0	0	0	0	0	1	0	1	0	0	0	0	0	1	0	0	0	MDR
18	0	0	0	0	0	0	0	1	0	1	0	1	0	0	0	1	1	0	0	0	0	MDR
19	1	0	0	0	0	0	0	0	0	1	0	1	0	0	0	1	0	0	0	0	0	MDR
20	1	1	0	0	0	0	0	1	0	1	0	1	1	0	1	1	0	0	0	0	0	MDR
21	1	0	0	0	0	1	0	1	0	1	1	1	1	0	0	1	1	0	0	0	0	MDR
22	1	1	0	0	0	0	0	1	0	1	1	1	0	0	0	0	0	0	0	0	0	MDR
23	1	1	0	0	0	0	0	1	0	1	0	1	1	0	0	1	0	0	0	0	0	MDR
24	1	1	0	0	0	0	0	1	1	1	1	1	1	0	0	1	0	0	0	0	0	MDR
25	1	1	0	0	0	1	0	1	0	1	0	1	1	0	0	1	0	1	0	0	0	MDR
26	1	1	0	0	0	0	0	1	0	1	0	1	0	0	0	1	1	0	0	0	0	MDR
27	1	1	1	1	1	1	0	1	1	1	1	1	1	0	1	1	0	0	1	0	0	XDR
28	1	1	0	0	0	1	0	1	0	1	1	1	1	0	0	1	1	1	0	0	0	MDR
29	1	1	1	1	1	1	0	1	0	1	1	1	1	0	1	1	0	1	0	0	0	MDR
30	0	0	0	0	0	1	0	1	0	1	1	1	0	0	0	1	1	1	0	0	0	MDR
31	0	1	1	1	1	1	0	1	1	1	1	1	1	0	0	1	0	0	0	0	0	MDR
32	1	1	1	1	1	1	0	1	0	1	1	1	1	0	0	1	1	0	0	0	0	MDR
33	1	1	0	0	0	1	0	1	0	1	0	1	1	0	1	1	1	0	0	0	0	MDR

34	1	1	1	1	1	1	1	1	0	1	1	1	1	0	0	1	1	1	0	0	0	MDR
35	1	1	1	1	1	1	1	1	1	1	1	1	1	0	0	1	0	0	0	1	0	XDR
36	1	1	1	1	1	1	0	1	1	1	1	1	1	0	1	1	0	0	0	0	0	MDR
37	0	0	0	0	0	1	0	1	0	1	0	0	0	0	0	1	1	0	0	0	0	MDR
38	0	1	0	0	1	1	0	1	0	1	0	0	0	1	1	1	1	0	0	0	0	MDR
39	1	1	1	1	0	0	0	1	0	1	1	1	1	0	0	1	1	1	0	1	0	MDR
40	0	0	1	1	1	0	0	1	0	1	1	1	1	1	1	1	1	1	0	1	0	MDR

Amgs: Aminoglycosides; **Fqns:** Fluoroquinolones; **Sphs:** Sulphas; **Nts:** Nitrofurans; **Pncn:** Penicillin; **2nd Cps:** 2nd Generations Cephalosporins; **3rd Cps:** 3 rd Generations Cephalosporins; **4th Cps:** 4th Generation Cephalosporins; **Mnbm:** Monobactams; **β-lactams + Inh:** β-lactams + Inhibitors; **tyn:** Tetracyclin; **Cpnm:** Carbapenems. **1:** Resistant phenotype; **0:** Susceptible phenotype
